# Sagittal imbalance of the spine is associated with poor sitting posture among primary and secondary school students in China: a cross-sectional study

**DOI:** 10.1186/s12891-022-05021-5

**Published:** 2022-01-28

**Authors:** Li Wen, Xiang Lin, Chaoqun Li, Yuqi Zhao, Zhenghui Yu, Xu Han

**Affiliations:** 1 School of Sports and Health, Nanjing Sport Institute, Nanjing, Jiangsu China; 2 Department of Computer Science, New Jersey Institute of Technology, Newark, NJ USA; 3 School of Kinesiology, Shanghai University of Sport, Shanghai, China; 4 School of Social Sports and Health Sciences, Tianjin University of Sport, Tianjin, China

**Keywords:** Children, Student, Posture, Spine, Sagittal angle of the spine, Kyphotic deformity

## Abstract

**Background:**

Long-term poor posture may affect the morphological development of the spine. However, there is no definite answer as to how writing posture affects students’ spine. This study attempted to compare the sagittal curvature of the spine between sitting and standing postures in adolescents to reveal the variation rule of spinal sagittal curvature of students with learning posture, and to discover the key factors that may affect students’ spinal health.

**Methods:**

1138 participants (male, 604; female, 534; age range, 6–18 years) from three schools in Tianjin, China, including 570 primary school students and 568 secondary school students. This study used SpineScan and PA200 Station Posture Assessment System to assess the sagittal curvature of the spine for three postures: sitting on a chair in upright position, seated at a desk while reading/writing, and standing in natural relaxed position. Analyze the difference between spine angle of the three postures and the correlation between the sagittal plane angle of the spine and body posture.

**Results:**

The mean sagittal angle of the spine changed when the participants were in reading/writing position compared to standing position, with the lumbar lordosis angle significantly decreased (*p* < 0.05) and the thoracic kyphosis angle significantly increased (*p* < 0.05). The TKA and LLA angles were abnormal in 33 and 52% of students in reading/writing posture respectively. There was a significant correlation between sitting posture and standing spinal Angle and were positively correlated with the height of the teenager (*p* < 0.05). By contrast, a higher percentage of TKA and LLA subjects in the standard reading/writing posture reference range maintained normal spinal shape while standing.

**Conclusions:**

The angle of thoracic kyphosis significantly increased from standing posture to upright sitting, reading/writing posture, while lumbar lordosis significantly decreased or even disappeared. There was a significant correlation between sagittal angle of spine in different postures. The poor sitting posture associated with sagittal angle abnormalities impact the shape of the spine such that sagittal imbalance was also observed when students in natural standing posture. Height is an important factor affecting the sitting spine shape of students.

## Background

The spine constitutes the central axis and pillar of the human torso. To relieve vertical pressure, the human spine has evolved four physiological curves in the sagittal plane that are a biomechanical masterpiece [1, 2]. In addition to the shape of the cone and intervertebral disc of the spine itself, the muscles acting on the spine related to the maintenance of posture also play an important role in the physiological curves of the spine. Unbalanced contraction of muscles antagonizing each other in function will lead to changes in the physiological curvature of the spine [3]. The physiological curvature of the spine is closest to the natural state that reduces energy consumption and maintains the curvature in the most relaxed state in a natural standing position. By contrast, the balance of the spinal curvature is pulled by the muscles and deviates from this relaxed state in sitting position, resulting in excessive kyphosis of the thoracic vertebrae and a reduction or disappearance of lumbar lordosis [4, 5]. Existing studies have shown that sitting for a long period greatly changes the tension of the body’s soft tissues [6]. Prolonged poor sitting posture over time is a root cause of spinal abnormalities. This is exacerbated among people, for example students, who use desks and chairs unsuited to their height [7, 8].

The change in body posture that deviates from the normal state is called a poor posture and is one of the most serious problems related to normal physical development in children [9, 10]. Children with poor posture have a higher frequency of headache, cervical and lumbar pain [11]. The skeletal systems of young school-age children are in the developmental stage, and their spines are characterized by rapid growth and high susceptibility to diseases caused by external factors [12, 13]. During this period, the stabilization of the anterior and posterior curvatures of the spine has not been completed. When children begin attending school, their lifestyle is markedly changed, as they are required to spend more time sitting at desks and chairs. Long-term incorrect posture beginning at this stage may affect the morphological development of the spine [14], further leading to Scheuermann’s Kyphosis and impair lung function [15, 16].

Few studies have examined the effect of poor reading/writing posture on the sagittal shape of the spine in children and teenagers or investigated the differences in the sagittal shape of the spine among teenagers associated with different sitting positions. To address these issues, the following hypothesis are proposed:

(1) Poor reading/writing posture in teenagers can cause their spines to deform.

(2) Students whose desks and chairs don’t match their height may be at higher risk of spinal deformity.

## Methods

### Study design

In this cross-sectional study, spinal angle in three position and body posture data were collected from 1138 students. Patients who were not diagnosed as AIS, underwent surgery and were not in the age range of 6–18 were excluded. Ethics was approved by the institutional review board of Tianjin University of Sport, Tianjin, China (approval number TJUS2017015). All participants and their guardians understood the purpose and method of this study.

### Participants

The study participants were from three schools with middle and primary schools in Tianjin, China, and randomly selected a class from each grade of each school for measurement.

### Study parameters

The thoracic spine kyphotic angle (TKA) and the lumbar lordosis angle (LLA) of all participants were measured using the SpineScan (OrthoScan Technologies, Rosh Pina, Israel), a digital preprogrammed inclinometer that assesses the spinal curvature by sliding it down the spine. It is a small user-friendly instrument, does not require much training, and spine evaluation with it takes less than a minute [17]. This convenient device is more efficient and suitable than using an imaging measurement method for a study with a large sample size.

During the measurements, the students were asked to stand naturally and then to sit on the chairs used in their classes. The students were asked to straighten their backs, and the curvature for the upright sitting position was immediately measured. They were then asked to remain seated at their desk and to copy a paragraph of text. Measurements of the spinal curvature at the desk during reading/writing were obtained after 5 min. Each posture was measured three times, and the averages of each of the three results were calculated.

The measurements of the desks and chairs used by the students in this study followed the GB/T3976–2014 Standard of Functional Dimensions and Technical Requirements of School Table and Chair proposed by the General Administration of Quality Supervision, Inspection and Quarantine of China [18]. Thus, primary and secondary school students have different sized desks and chairs. The heights of desks and chairs used by secondary school students (grades 7–9) are 700 (±2) mm and 400 (±2) mm, respectively, whereas those used by primary school students (grades 1–6) are 610 (±2) mm and 340 (±2) mm, respectively.

The height and weight of each participant were measured used Height and weight scale ALN-300 (AnLiNuo China). The vital capacity of the participants was measured using the FGC-A+ Lung Function Instrument (AnKe, China). They were asked to breathe in as much air as they could and then to forcefully exhale through the blower as hard as they could, blowing for three consecutive 15-s intervals. The pelvic inclination and neck inclination of participants’ standing postures were measured using a PA200LE Station Posture Assessment System (Big Sports, Japan). The equipment can measure the left-side and right-side postures of subject distinguished by the median line. In this study, angle of pelvis (angle between the anterior superior iliac spine and the posterior superior iliac spine) and neck (angle between C2 and C7 spinous processes) on the left and right sides were measured and averaged.

### Statistical analysis

Logistic regression analysis was used to analyze the relationship between age and sagittal angle of spine in standing position each gender. One-way ANOVA was used to analyze the difference of sagittal angle between the three postures, Bonferroni adjustment was used for post-hoc analysis. Independent-samples *t-*test was used to analyze the differences in lung capacity and standing spinal angle between the subjects with normal and abnormal spinal morphology in reading/writing position. Pearson’ correlation coefficients was used to test the correlations between variables, including sagittal angle of the spine in different postures, pelvic and neck inclination and spine angle, height and spine angle in reading/writing posture. Two-side *p* values < 0.05 were considered statistically significant. All statistical tests were conducted using SPSS (v23.0).

## Results

A total of 1138 students participated were included in the study, including 534 females and 604 males. There were 570 primary school subjects and 568 junior middle school subjects. Due to the different school age of students, the age of students in the same grade is different, the age range of these teenagers is between 6 and 18 years old.

Participants were grouped according to their age, one group for each age from 6 to 18 years old, and the mean values of TKA and LLA were calculated for each age group, by sex. A regression analysis of age and sagittal angle of spine in standing position each gender found that the TKA and LLA angles of female adolescents changed significantly with age, but males did not (Table 1). These descriptive data appeared to show that TKA in male participants was generally higher than that of females of the same age, and both male and female participants’ thoracic curves seemed to have a small growth spurt at age 11(Table 2).

**Table 1:** Regression analysis of age and sagittal angle of spine in standing position

Variable	Sex	Sample size	Β (95% CI)	*P*
TKA	Male	604	−0.141(−0.348–0.065)	0.180
	Female	534	−0.420(− 0.661–-0.178)	0.001^**^
LLA	Male	604	0.016(−0.232–0.263)	0.901
	Female	534	−0.415(− 0.737–-0.093)	0.012^*^

* *p* < 0.05, ** *p* < 0.01

**Table 2:** Mean thoracic kyphosis angles (TKAs) and lumbar lordosis angles (LLAs), by sex and by age

Sex	Age (years)	Sagittal angle of spine in natural standing position	
		TKA (°)	LLA (°)
Male	6	26.286(7.297)	−32.500(9.504)
	7	34.583(9.053)	− 27.167(12.350)
	8	37.372(9.722)	− 24.488(9.480)
	9	36.797(8.944)	− 23.678(9.659)
	10	35.868(8.731)	−24.015(9.770)
	11	38.341(7.886)	−27.073(12.507)
	12	35.364(8.355)	− 25.697(9.554)
	13	31.383(5.968)	−28.243(8.667)
	14	32.747(5.821)	−26.670(8.352)
	15	34.273(5.147)	−25.955(6.433)
	16	33.412(6.663)	−25.618(9.912)
	17	31.500(6.658)	−23.750(9.912)
	18	43.000(5.657)	−27.500(3.536)
Female	6	27.906(7.731)	−24.437(17.729)
	7	30.833(8.809)	−23.738(12.057)
	8	34.463(11.227)	−24.439(10.789)
	9	33.939(10.509)	−23.653(8.430)
	10	37.527(10.059)	−24.964(10.173)
	11	37.119(8.674)	−25.619(10.138)
	12	32.333(8.222)	−26.361(19.722)
	13	28.247(6.134)	−26.194(13.193)
	14	28.393(5.995)	−28.085(9.582)
	15	30.423(4.884)	−26.462(7.495)
	16	29.377(6.968)	−26.943(9.141)
	17	24.000(9.539)	−34.667(7.234)
	18	32.000(9.607)	−36.000(4.950)

Data represent mean (standard deviation)

The average TKA values in upright sitting position is smallest, increase when standing naturally, and reach a maximum in reading/writing position. The absolute value of LLA is maximum in natural standing position. Many teenagers have lumbar lordosis disappear when they sit down, resulting in lumbar kyphosis. Significant differences were observed in TKA and in LLA for the three different postures (Table 3).

**Table 3:** Average thoracic kyphosis angles (TKAs) and lumbar lordosis angles (LLAs) for three postures in all participants

Angle Measured	Body Posture		
	Natural Standing	Upright Sitting	Seated Reading/Writing
TKA	32.873 (8.669)^#^	25.138 (8.729)^*^	33.529 (9.706)^#^
LLA	−26.043 (10.615)^#^	−12.661 (9.063)^*^	0.772 (12.676)^*#^

Numerical values represent the mean (standard deviation)

*p < 0.05 compared with natural standing position; #*p* < 0.05 compared with upright sitting position

### Thoracic kyphosis angle

**Fig. 1:**
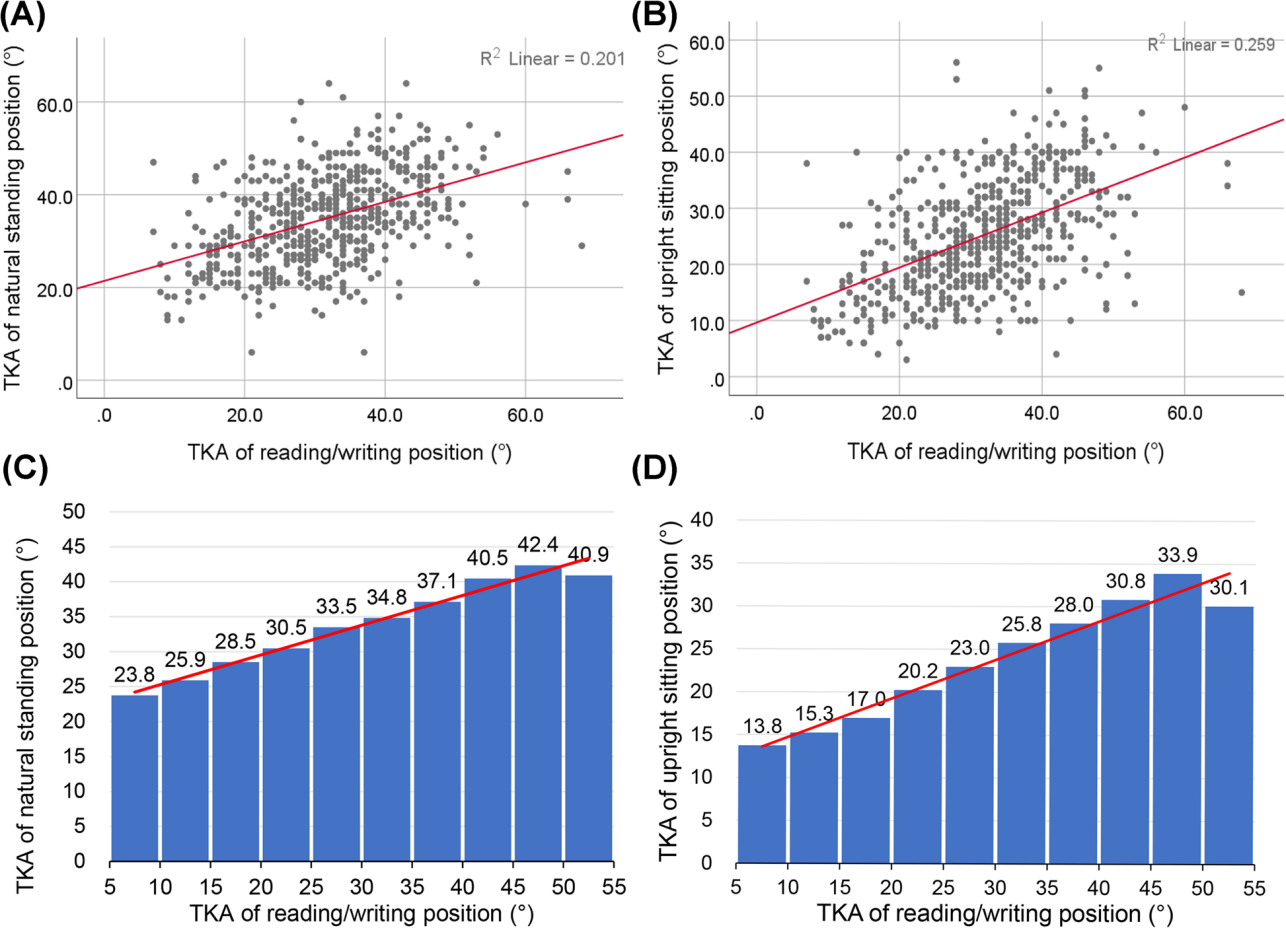
Correlations and histograms of thoracic kyphosis angles (TKAs) in the seated reading/writing position vs. the natural standing position or an upright sitting position in primary school children

**Fig. 2:**
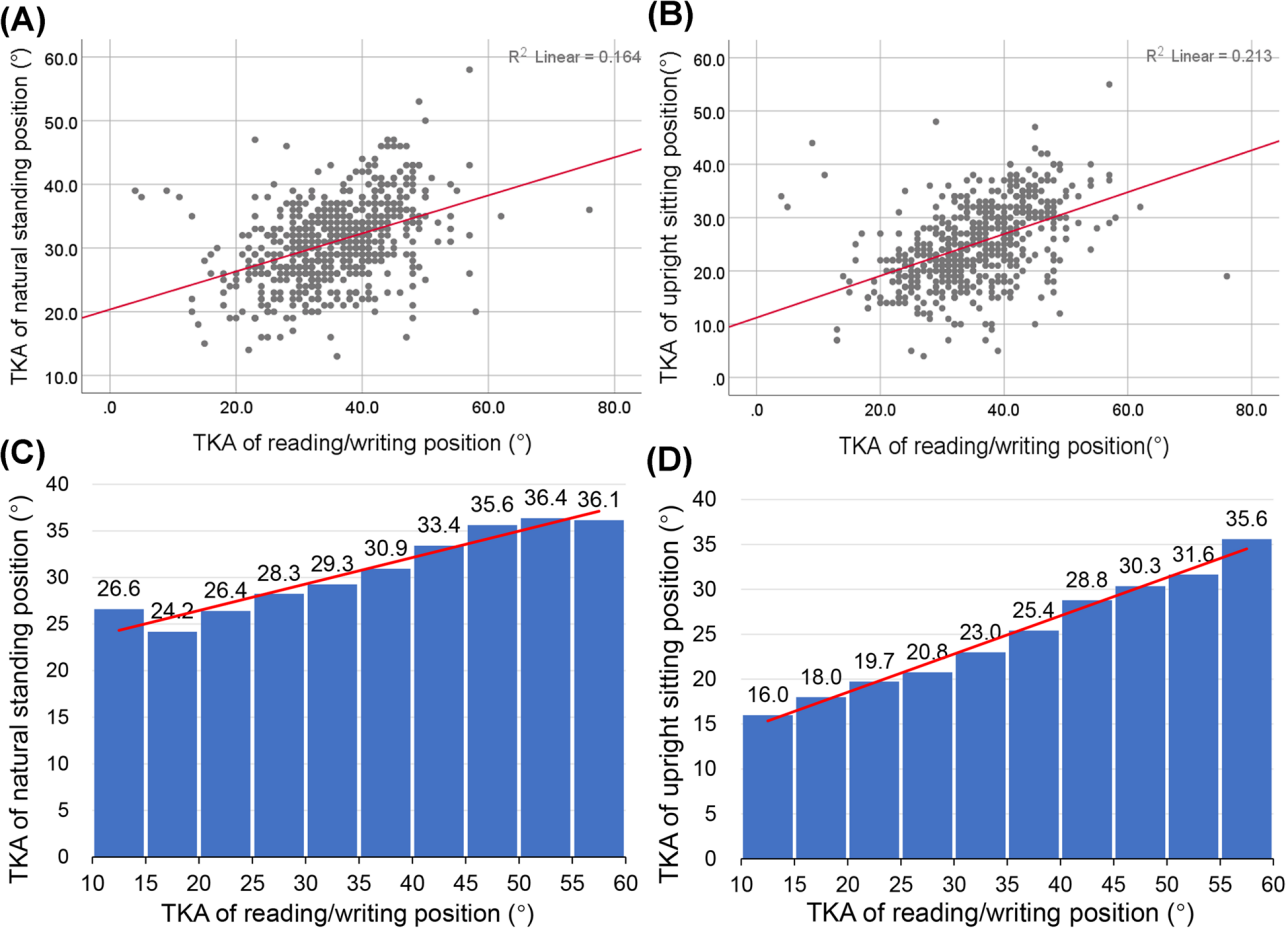
Correlations and histograms of thoracic kyphosis angles (TKAs) during reading/writing vs. a natural standing position or vs. an upright sitting position in secondary school students

As shown in Table 3, participant TKAs were significantly different for the three postures. In correlation analyses found that the TKAs for the reading/writing posture were significant linear relationship with the TKAs of the natural standing posture among 570 primary school (R^2^ = 0.201, *p* < 0.01, Beta = 0.426, 95%CI = 0.356–0.496) and 568 secondary school participants (R^2^ = 0.164, *p* < 0.01, Beta = 0.299, 95%CI = 0.243–0.354) (Figs. 1 and 2). The results of correlation analysis indicated that the read/writing position TKA is associated with the thoracic curve in participants when they stand naturally.

It is generally believed that the standard TKA should be 20° to 40°, with angles greater than 40° or less than 20° considered excessive flexion and extension of the thoracic vertebrae [19, 20]. Based on normal and abnormal TKA, participants were divided into normal (20° ≥ TKA ≥ 40°) and abnormal (TKA < 20° or > 40°) subgroups. We found the significant differences in the TKA of natural standing posture between the two subgroups (*p* < 0.01), and 244 people (21%) had abnormal TKA in standing posture and 364 people (32%) had abnormal reading/writing posture. When the TKA during reading/writing was in the standard reference range, then 83.1% of the participants had TKAs during standing in the normal reference range; conversely, when the TKA during standing was in the standard reference range, then 71.9% of the participants had TKAs during reading/writing in the normal reference range.

A linear correlation was found between the TKAs for the postures during reading/writing and the upright sitting position among primary school (R^2^ = 0.259, *p* < 0.01, Beta = 0.490, 95%CI = 0.422–0.558) and secondary school participants (R^2^ = 0.213, *p* < 0.01, Beta = 0.393, 95%CI = 0.330–0.455) (Figs. 1 and 2). This result indicated that secondary students with thoracic vertebrae that are too kyphotic in the reading/writing position have difficulty reaching the normal thoracic curvature angle when they sit upright.

### Lumbar lordosis angle

A significant difference was found in LLAs among the three postures in primary and secondary school students. Most participants were able to maintain lumbar lordosis when standing naturally or sitting upright, but LLA was absent and lumbar kyphosis occurred in the reading/writing position (Table 3).

LLAs for the three postures was stratified according to whether lumbar lordosis could be maintained [21]. We found that almost all subjects (1139, 99%) were able to maintain lumbar lordosis while standing, but half of the students (590, 52%) suffer lumbar kyphosis while studying. There was a significant difference in the natural standing LLA for students who maintained vs. those who lost LLA during seated reading/writing (*p* > 0.05). These results suggested that there was a significant interaction between the seated reading/writing position and the natural standing LLA such that students who maintained standard reference LLAs in a seated reading/writing position had a healthy lumbar curvature when standing naturally, and vice versa.

Proper thoracic Angle also helps maintain lumbar lordosis. Among all participants, 93.7% of the 774 students who were able to maintain the standard reference range for the TKA in seated reading/writing position could also maintain normal lumbar lordosis.

### Association between thoracic curvature and vital capacity

Studies have shown that body posture affects respiratory function and the range of motion of the chest and diaphragm [22].

**Fig. 3:**
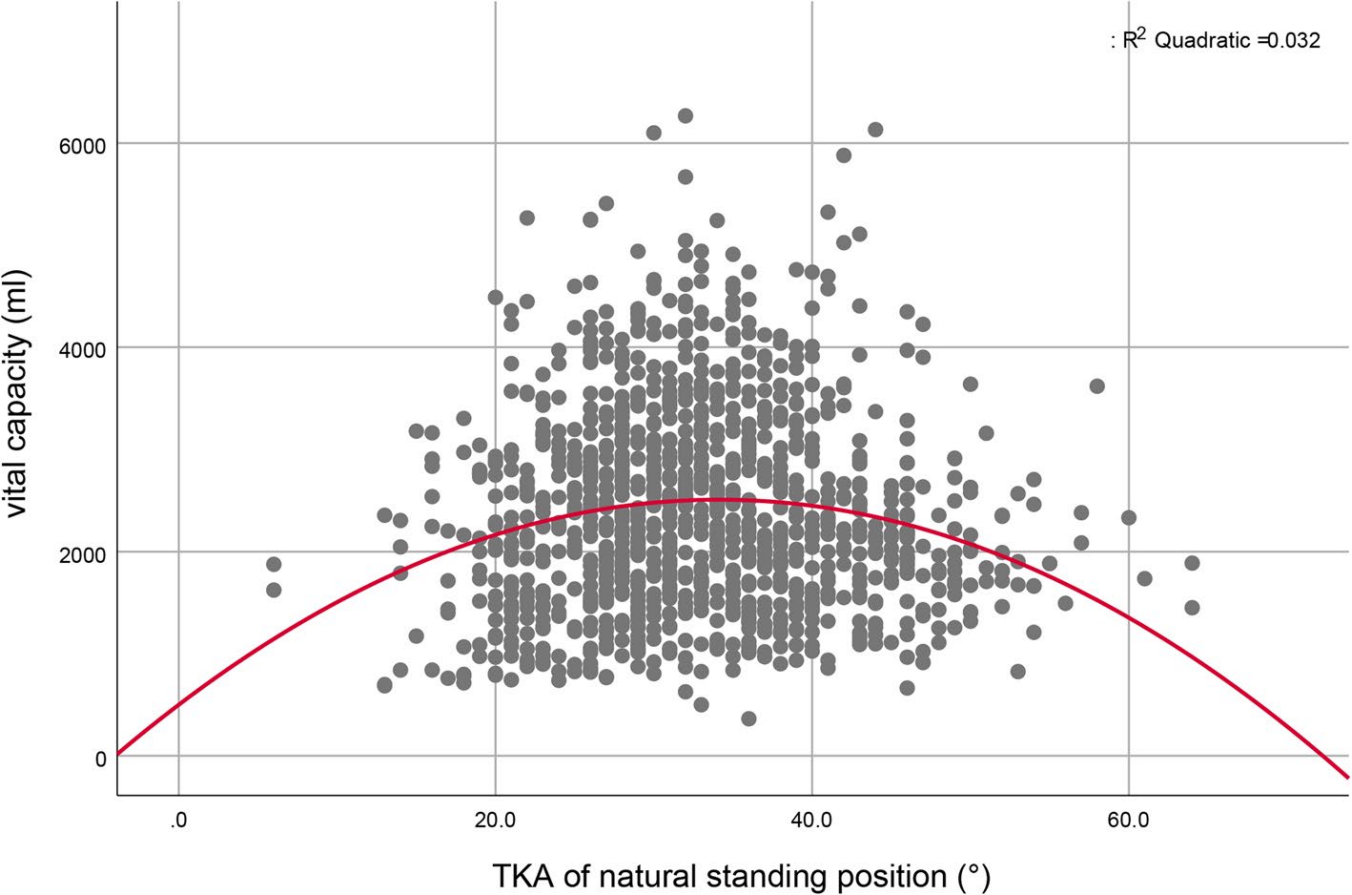
Scatter diagram of the association between the natural standing position thoracic kyphosis angle (TKA) and vital capacity

A scatter plot of the association between TKA during natural standing and vital capacity (Fig. 3), shows an obvious inverted U shape. Using independent-samples t test analyses found that the vital capacity of students with a normal TKA (defined as between 20° and 40°) was significantly higher than that of students with a greater or lesser TKA (*p* < 0.01).

### Body posture and sagittal plane angle of the spine

**Fig. 4:**
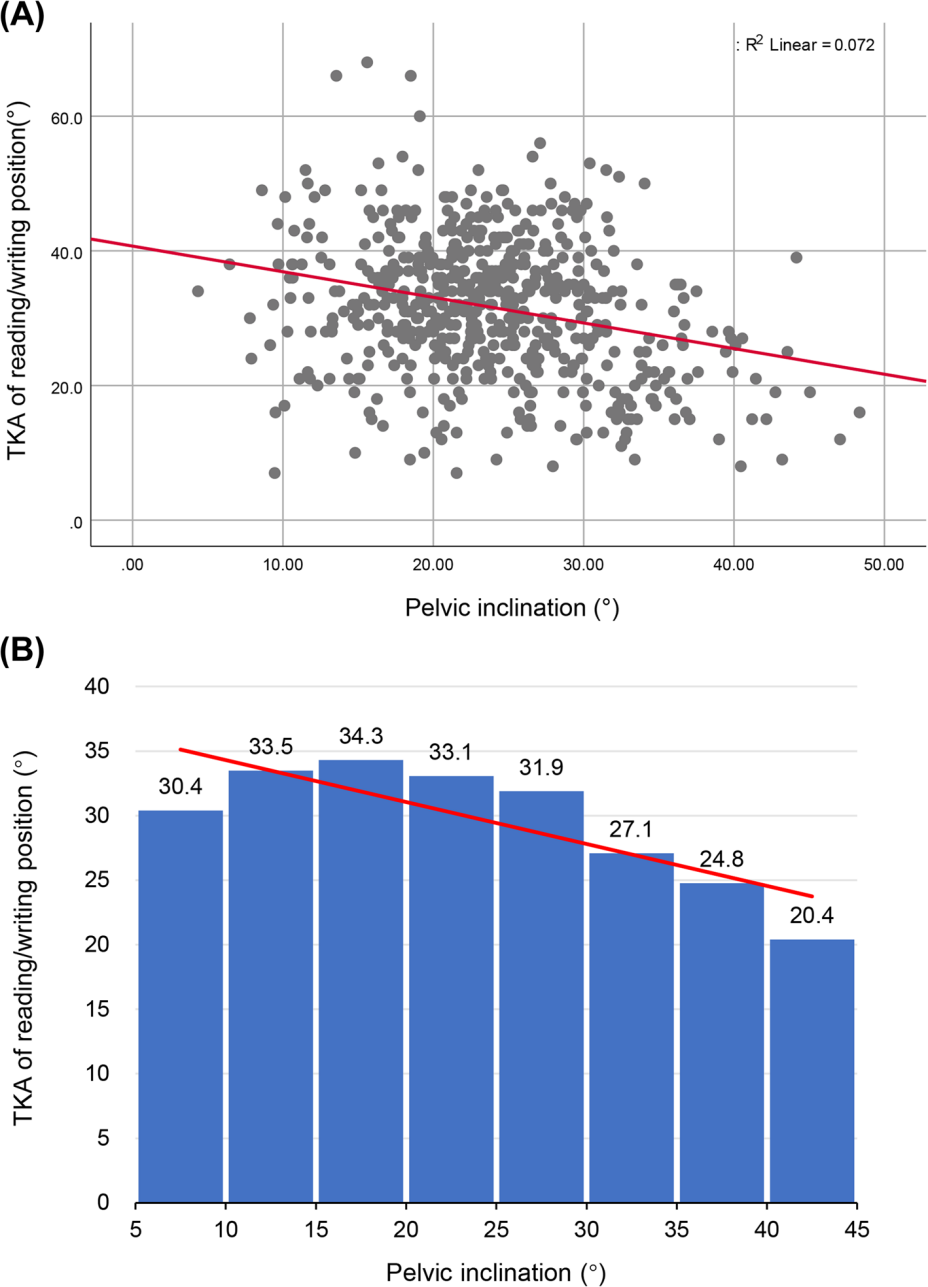
Scatter plot and histogram showing the association between pelvic inclination and seated reading/writing thoracic kyphosis angle (TKA) in primary school children

**Fig. 5:**
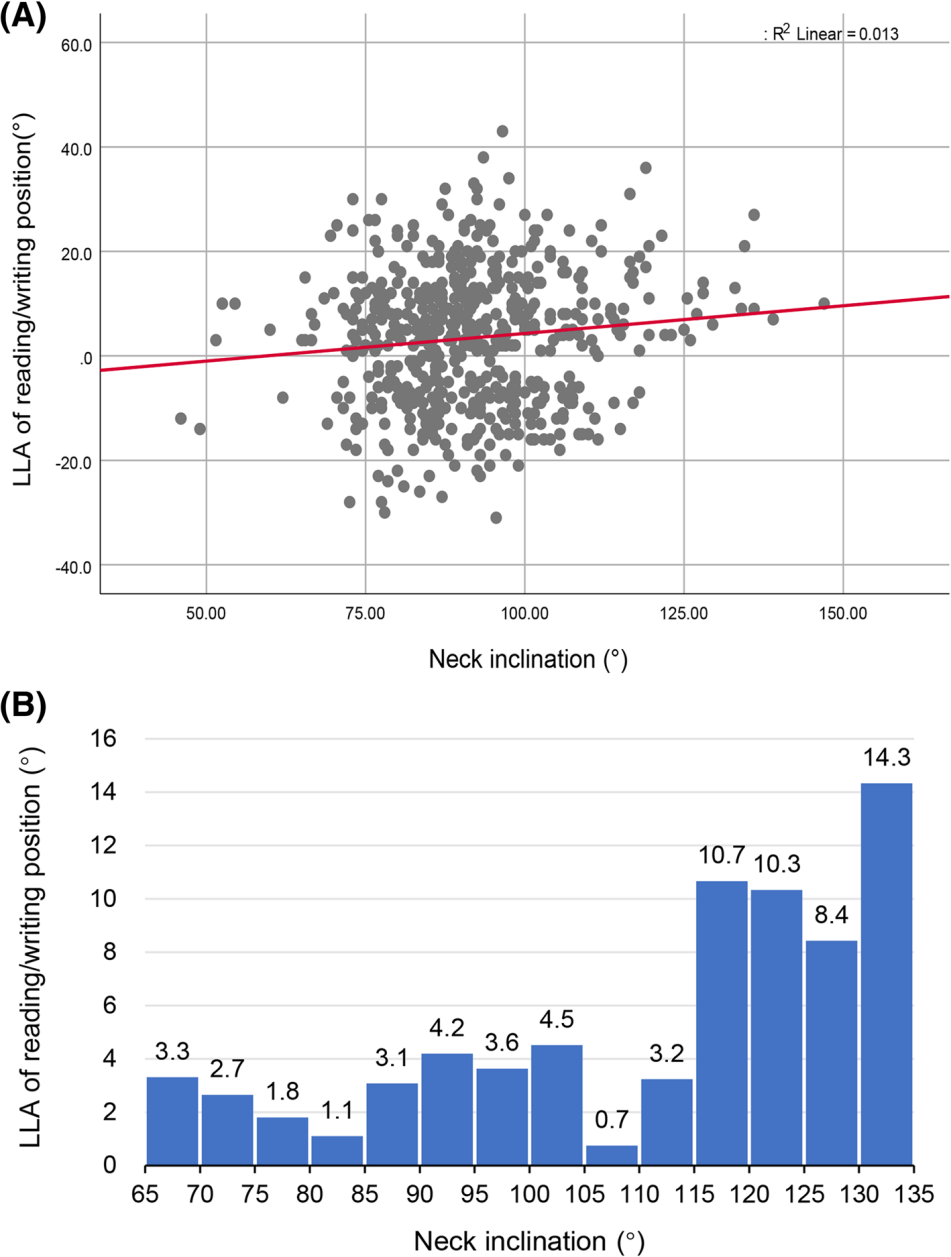
Association between neck inclination angle and seated reading/writing lumbar lordosis angle (LLA) in primary school children

Body posture, as indicated by the pelvic inclination and neck inclination of the natural standing posture, was measured using a PA200 Station Posture Assessment System. We found a significant linear association between the inclination angle and the pelvic TKA of the seated reading/writing position in primary school participants (R^2^ = 0.072, *p* < 0.01, Beta = − 0.381, 95%CI = -0.493–-0.268) (Fig. 4). The correlation was not significant among secondary school student. Moreover, there were significant correlations between the LLA of the seated reading/writing position and the inclination of the neck in primary school students (R^2^ = 0.013, *p* < 0.01, Beta = 0.106, 95%CI = 0.030–0.182) (Fig. 5).

### Height and sagittal plane angle of the spine

**Fig. 6:**
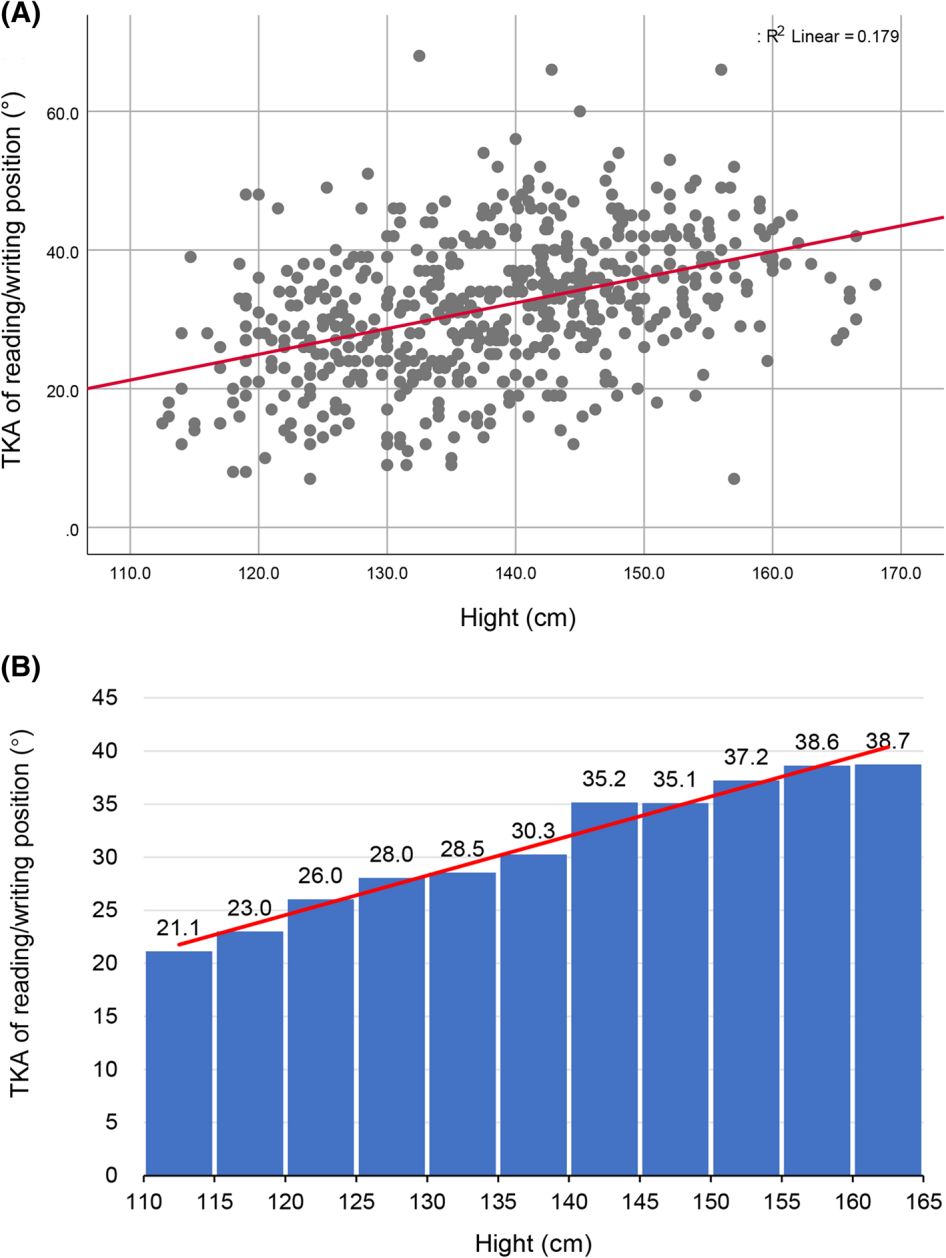
Scatter plot and histogram showing the association between and seated reading/writing thoracic kyphosis angle (TKA) and height in primary school children

**Fig. 7:**
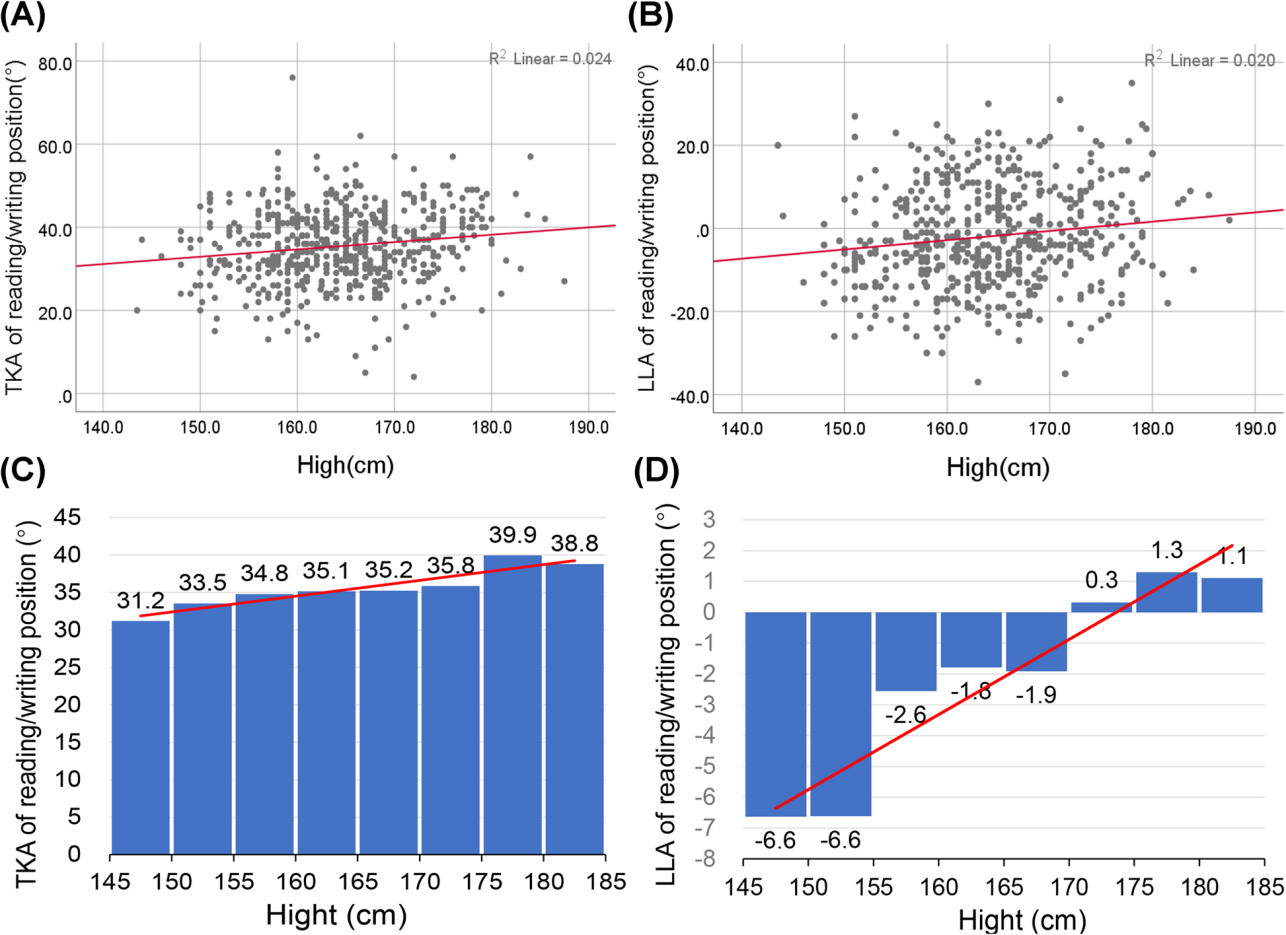
Scatter plots and histograms showing the associations of seated reading/writing thoracic kyphosis angle (TKA) and lumbar lordosis angle (LLA) with height in secondary school students

We found significant correlations between the height and their seated reading/writing TKAs among primary school students (R^2^ = 0.179, *p* < 0.01, Beta = 0.371, 95%CI = 0.305–0.436) (Fig. 6), and height is significantly associated with TKA (R^2^ = 0.024, *p* < 0.01, Beta = 0.177, 95%CI = 0.084–0.270) and LLA(R^2^ = 0.020, *p* < 0.01, Beta = 0.223, 95%CI = 0.096–0.351) (Fig. 7) in secondary school subjects. To further assess the effect of height on the shape of the spine in these students, the value of TKAs and LLAs by 5-cm increments in height were analyzed. Figures 6 and 7 show significant positive correlations between TKA and height and between LLA and height in secondary school students. Lumbar curvature began to change from lordosis to kyphosis once individuals were taller than 170 cm, suggesting that the desks and chairs they were used are no longer suited to their height. The thoracic curvature also increased linearly with height in primary school children, but their thoracic kyphosis angles were within the normal reference range. Although there was no linear association between lumbar curvature and height in primary school children, almost all the students had lumbar kyphosis when reading and writing at their desks.

## Discussion

The movement of the torso in the sagittal plane is mainly undertaken by both muscles and vertebrae of the neck, chest and waist. When in the natural standing position, the muscles used to maintain the physiological curves in the spine are in the most relaxed state. The physiological curvature of the spine is also closest to the natural state when the body is very young, but the spine is prone to pathological deviations under prolonged improper posture [2, 23, 24]. The study involved 1138 primary and secondary school students. We investigated the changes in spinal morphology induced by poor posture in adolescents and analyzed the influencing factors.

Our study found that comparing natural standing to an upright sitting posture, the physiological curvature of the spine is greatly changed in the sitting position, resulting in a tendency for increase in thoracic curvature, and loss of lumbar kyphosis. The thoracic curvature, a more stable segment of the spine, has a smaller angle of motion and thus is easier to maintain [25], whereas the lumbar curvature, a more flexible segment, has a larger range of motion [2]. Although the best LLA is unclear, maintaining lumbar lordosis is of substantial physiological significance [26], with many studies showing that abnormal lumbar curvature can lead to nonspecific low back pain [27, 28]. With increasing sagittal changes of the spine, angle changes and loss of lordosis bring a greater load to the lumbar spine. Previous imaging evidence has shown that when a person sits in a relaxed posture on a stool, the entire lumbar lordosis below the lumbar L1–L2 level becomes more kyphotic, and lumbar lordosis at L4–L5 and the lumbosacral joint L5–S1 levels decreases significantly. Relaxation of the sacrospinalis and multifidus muscle of the waist, which maintain lumbar lordosis, increases pyramidal pressure at this location [29].

Notably, there was a strong linear correlation between TKA and LLA in sitting and standing posture, suggesting that excessive spinal flexion in poor sitting posture could impair standing position. It also implied that maintaining reading/writing posture within the standard references range can relieve spinal deformation. Students spend a lot of time reading and writing every day, and when they relax their postural control, the spine becomes out of balance, which may cause pathological changes [30]. Previous studies have found that longer periods of time may increase the chances of inappropriate posture [31], which worsens the spinal health of teenagers, and several recent studies have reported similar results [32].

Our results showed that the standing pelvic angle was significantly correlated with TKA, and the neck inclination angle was significantly correlated with LLA. Because the range of motion of the sacroiliac joint is very small, the pelvic inclination measured by the PA200 system was similar to the sacral tilt angle of the participants [33]. The human pelvis gradually tilts forward from sitting to standing [34, 35],thus, the sitting posture has a lower sacral inclination angle than the standing posture. We speculate that the decrease of the pelvic oblique angle during seated reading/writing leads to decreased lumbar lordosis and even to lumbar kyphosis. When lumbar support is reduced or absent, thoracic kyphosis will increase accordingly [36]. Students seated improperly at a desk for a long time may experience increased kyphosis, and TKA increases substantially, exceeding the normal kyphosis angle range of 20° to 40°. At the same time, to maintain the distance between the eyes and the reading/writing material, the cervical vertebra in the sagittal plane adapts as a whole as a compensatory mechanism for maintaining horizontal gaze. In this cross-sectional study, kyphosis was observed in a high proportion of adolescents, suggesting a serious spinal health problem for adolescents.

Most secondary school students who sit at chair/desk combinations that are too small for their height are unable to maintain a good sitting posture. When they continue in this position in order to see the word, high-intensity pressure will inactivate the local spinal stabilizing muscles that resist fatigue and increase tension on the connective tissue. The shape and posture of the spine will become set, even when the student is standing naturally. In addition, excessive anterior traction of the neck increases the load of the muscles in the shoulder and neck and induces upper cross syndrome [37, 38], while the loss of lumbar lordosis is the direct cause of low back pain [36, 39]. Excessive changes in thoracic spine curvature can also affect pulmonary ventilation (Fig. 3). Culham et al. compared the vital capacity of normal women and women with thoracic kyphosis caused by osteoporosis and found that vital capacity and rib mobility were significantly impaired in women with thoracic kyphosis [40]. Therefore, a normal chest curve is essential to the volume of the chest and the ventilatory function of the lungs [22, 41]. Our conclusions confirm and extend those of that study. The effect of the chest curvature on vital capacity was substantial, even when no pathological thoracic kyphosis was present, vital capacity was significantly changed with TKA. By contrast, we found that participants who were able to maintain TKA and LLA within standard references ranges while seated reading/writing at their desks had better spine shape while standing. This highlights the importance of a good sitting position.

The analysis of height and spine morphology reflects the hidden spinal issue among secondary school students: the taller the students, the more likely they are to stoop and hunch. When children enter puberty rapidly, some may grow tall faster than their peers, leading to a greater difference in their heights. Thus, desks and chairs that are only one size are not suitable for the various heights of students. Taller students must use more body flexion in a desk that is too small to maintain the correct distance from a textbook to clearly see it. This flexion is accomplished not only by increasing thoracic curvature but also by reducing lumbar lordosis. This is reflected in the participants’ mean TKAs and LLAs (Table 3). However, most primary and secondary schools in China do not have the ability to purchase desks and chairs based on the various heights of students.

If the pelvis is rotated forward during development while sitting, sciatic support is reduced, lumbar support is strengthened to obtain neutral lordosis of the lumbar spine, and the thoracic cavity is relaxed. These changes cause synergistic activation of the superficial lumbar multifidus muscles and internal oblique muscles, resulting in local stabilization of the lumbosacral region without a high compressive load [28, 29]. However, most primary and secondary schools in China have desks and chairs that are not equipped with supporting cushions for the lumbar vertebrae and thus do not provide stable support for students’ lumbar curvature.

The majority of studies on the morphology of the spine have focused on the coronal plane, with few studies assessing the sagittal plane of the spine in children and teenagers [42]. As teenagers spend increasingly more time sitting, the deformation of their spine in the sagittal plane is becoming a more common and noteworthy problem. Our results suggest that it is important to remind students to always sit well during learning. Reducing students’ sedentary time or providing students with desks and chairs suitable for their height or that maintain their sacral oblique angle may have a positive effect on improving the sitting posture among teenagers in China.

## Conclusion

When primary and secondary school students reading/writing, thoracic spine kyphotic angle increased significantly and the lumbar lordosis angle decreased significantly and even kyphosis appeared. The sitting posture is highly correlated with the sagittal angle of the spine of standing and upright position. Poor sitting posture will change subjects’ spine shape. We speculate that this is the result of the body’s re-adaptation which was caused by long-term deviation of the spine shape. When students use desks and chairs with the same height, the height of the students becomes an important factor affecting the shape of teenagers’ sitting spines. Taller students’ spines need more flexion and are more prone to imbalance. Therefore, the use of desks and chairs with appropriate height or lumbar support may be conducive to maintain proper spinal shape and alignment.

### Limitations

Some limitations in this study should be considered in interpretation of the findings and construction of future studies. In this study, spinal angle data were all measured with a hand-held measuring ruler. Due to the lack of imaging evaluation, the changes of each segment of the spine cannot be visually observed. We found that students of different heights had different spines during learning, and speculated that this was due to inappropriate desks and chairs, how the effect of desks and chairs of different heights on subjects’ spinal angle has not been assessed. Future studies are required to solve these issues.

## Data Availability

The datasets used and/or analyzed during the current study are available from the corresponding author on reasonable request.

## Abbreviations

TKA: Thoracic kyphosis Angle
LLA: Lumbar Lordosis Angle
SD: Standard Deviation
CI: Confidence Interval
