# Correction to: Sagittal imbalance of the spine is associated with poor sitting posture among primary and secondary school students in China: a cross-sectional study

**DOI:** 10.1186/s12891-022-05303-y

**Published:** 2022-05-19

**Authors:** Chaoqun Li, Yuqi Zhao, Zhenghui Yu, Xu Han, Xiang Lin, Li Wen

**Affiliations:** 1 School of Sports and Health, Nanjing Sport Institute, Nanjing, Jiangsu China; 2 School of Kinesiology, Shanghai University of Sport, Shanghai, China; 3 School of Social Sports and Health Sciences, Tianjin University of Sport, Tianjin, China; 4 Department of Computer Science, New Jersey Institute of Technology, Newark, NJ USA

**Correction to: BMC Musculoskelet Disord 23, 98 (2022)**

**https://doi.org/10.1186/s12891-022-05021-5**

Following the publication of the original article [1] the authors reported the wrong sequence of authors in the author list. The correct author order should be: Chaoqun Li, Yuqi Zhao, Zhenghui Yu, Xu Han, Xiang Lin*, Li Wen*, declaring Dr. Xiang Lin as corresponding author together with Dr. Li Wen.

The original article [1] has been updated.

## References

[1] Li C, Zhao Y, Yu Z (2022). Sagittal imbalance of the spine is associated with poor sitting posture among primary and secondary school students in China: a cross-sectional study. BMC Musculoskelet Disord.

